# Pyrozone

**Published:** 1893-03

**Authors:** C. B. Atkinson


ARTICLE IV.

### PYROZONE.

---

The medicinal pyrozone is an aqueous solution containing three per cent. $H_2O_2$, which represents fifteen volumes of confined gas.

The former so-called fifteen-volume solutions are now claimed to be but ten volumes.

The range of use for this 3 per cent. pyrozone is similar to that in which the ordinary peroxide has been employed.

In this connection, I would like to say that diluted peroxide is often prescribed, and that I have never used it with any satisfaction when diluted, but always use it full strength.

Medicinal pyrozone is a good bleacher, but less penetrating in its effects than the stronger preparations.

The antiseptic pyrozone, containing 5 per cent. $H_2O_2$, or twenty-five volumes in ethereal solution, seems to be more generally serviceable in dentistry. The caution should be noted that concentration follows on evaporation of the ether. Thereby the antiseptic strength in time may become caustic. The effects of the 5 per cent. and of the 25 per cent., or caustic solution, are so similar in general character, that with the statement that the 25 per cent., in most instances, *is* caustic, though not in all, an indefinite, description as between the two will serve as an outline of the observations that have been had on these oxygen conveyors.

These ethereal preparations are neutral, and, though they decolorize litmus paper, it is by bleaching through the action of the oxygen on the organic coloring matter, not a reaction caused by acid.

They are inflammable and volatile. The first use made of them by me was in the bleaching of teeth. A

pledget of cotton soaked with pyrozone, 5 per-cent., was placed in a cavity, and the surface of the tooth wiped with the same strength. It produced a very considerable bleaching effect in a pulpless right-superior first bicuspid. This 5 per cent. strength has been used to remove the brown or green stain about the necks of children's teeth, care being exercised that the fluid does not touch the gum.

As a bleacher, pyrozone is (by its makers) advised to be used after applying an alkali.

Pyrozone alone bleaches teeth well. Whether it is a solvent of the organic substance of the tooth, or whether the chemical constitution of the tooth, being mainly alkaline, furnishes the basis for its activity, remains to be shown. Its application on soft tissue produces a bleaching effect strongly resembling an eschar. This progresses slowly after application, therefore caution should be exercised to give full time for this characteristic appearance before further application. The white, apparently coagulated surface resumes a pink tone slightly lighter than before the application of the pyrozone.

The change occupies from a quarter to one minute in whitening the surface, according to the character of the inflammation. The more the inflammation approaches suppuration, or the greater the congestion, the more rapid is the action of apparent coagulation. The fading of the white color occupies five to ten minutes.

Pyrozone in all strengths is a prompt hemostatic, though because of the caustic effect sometimes produced by both the stronger ethereal solutions, the 3 per cent. will probably best serve this office. Pyrozone acts more promptly on moist surfaces that on such as are dry. In a case of pyorrhea alveolaris, suppurating for eight months previous to treatment, the suppuration was controlled after one application of caustic pyrozone (25 per cent.) on all of the teeth except about the transverse processes between the right superior central and lateral, and the left inferior central and lateral. The processes themselves had softened, and will require further time to become restored to health. In this instance the caustic pyrozone caused a surface coagulation under which, or through which rather, the gas seemed to be liberated continuously, producing a crepitous sensation on passing the finger over the surface. This crepitation gradually subsided, the confined gas seemingly being given off through the external coagulated surface.

The color (white) fades more rapidly from the normal surface than from the abnormal, and as a diagnostic help pyrozone, 5 per cent., seems to have possibilities. Liquid alboline, benzoinol, or other *mineral-oil* product, as vaseline, will be found palliative to the prickling of the caustic.

It was intended to make some observations on phenata of cocaine, but the clinical opportunities ought for pyrozone have somewhat eclipsed the phenate. However, for the present it must suffice to state briefly that as a local anæsthetic for extraction, congested pulps, and in opening abscesses, it has proved a valuable aid, entirely subduing pain without injury.

The continued use of pyrozone has intensified the statements made, and with reference to the 5 per cent. or antiseptic pyrozone it has been found especially efficient in two somewhat grave cases, one of necrosis where the external plate of the alveolus of the inferior maxilla had been (in extraction) ruthlessly separated from the symphysis to the angle, the teeih of the entire lower denture having been ordered removed by a physician, who had externally poulticed an abscessed right inferior first bicuspid. Marchand's medicinal peroxide was also used in comparative tests with pyrozone (3 per cent. or medicinal), by injection in the sinuses. The resulting froth was in every instance thicker and more abundant with the pyrozone 3 per cent. than with the Marchand peroxide. This seems to indicate that the claim made by the maker of pyrozone 3 per cent., that their preparation *is* fifteen volumes and Marchand's but ten, is true. The abnormal tissue sooner returned to health, and the suppuration more rapidly ceases under treatment of pyrozone 3 per cent. than under Marchand's peroxide. In a case of severe ulceration of the left cheek, starting about the orifice of the duct of the parotid gland and extending to and slightly posterior to the condyle of the upper maxilla, the 5 per cent. strength of pyrozone has been employed in comparative tests with salicylic acid (saturated solution in alcohol). The pyrozone produced at first considerable pain, which rapidly subsided, leaving a clean non-suppurating surface. The salicylic acid produced pain for nearly six hours, and left a coagulum which in three days peeled off, leaving a tender surface with isolated spots of suppuration. The reduction in the depth of ulceration in both applications was evident, but the early relief from pain and the absence of the coagulum with the pyrozone 5 per cent. indicated it a preferable stimulant and antiseptic.

The great principle of cure is to remove abnormal conditions as nearly as possible, and let nature make the cure. In a general way, keep the body inside and out *clean*, and it will be healthy. The oxygen seems to effectively accomplish, and the apparent absence of all danger in its use places it in an enviable relation with other corrective agencies.—DR. C. B. ATKINSON, in *Cosmos*.

---

ARTICLE V.

